# Impact of the Vulnerable Preterm Heart and Circulation on Adult Cardiovascular Disease Risk

**DOI:** 10.1161/HYPERTENSIONAHA.120.15574

**Published:** 2020-08-17

**Authors:** Adam J. Lewandowski, Philip T. Levy, Melissa L. Bates, Patrick J. McNamara, Anne Monique Nuyt, Kara N. Goss

**Affiliations:** 1 From the Oxford Cardiovascular Clinical Research Facility, Division of Cardiovascular Medicine, Radcliffe Department of Medicine, University of Oxford, United Kingdom (A.J.L.); 2 Department of Pediatrics, Boston Children’s Hospital, Harvard Medical School, Harvard University, MA (P.T.L.); 3 Department of Health and Human Physiology (M.L.B.), University of Iowa; 4 Division of Neonatology (M.L.B., P.J.M.), University of Iowa; 5 Division of Cardiology (P.J.M.), University of Iowa; 6 Department of Pediatrics, Division of Neonatology, CHU Sainte-Justine, Faculty of Medicine, Université de Montréal, QC, Canada (A.M.N.); 7 Departments of Pediatrics (K.N.G.), School of Medicine and Public Health, University of Wisconsin-Madison.; 8 Medicine (K.N.G.), School of Medicine and Public Health, University of Wisconsin-Madison.

**Keywords:** cardiovascular diseases, hypertension, hypertension, pulmonary, premature birth, vascular stiffness

## Abstract

Preterm birth accounts for over 15 million global births per year. Perinatal interventions introduced since the early 1980s, such as antenatal glucocorticoids, surfactant, and invasive ventilation strategies, have dramatically improved survival of even the smallest, most vulnerable neonates. As a result, a new generation of preterm-born individuals has now reached early adulthood, and they are at increased risk of cardiovascular diseases. To better understand the sequelae of preterm birth, cardiovascular follow-up studies in adolescents and young adults born preterm have focused on characterizing changes in cardiac, vascular, and pulmonary structure and function. Being born preterm associates with a reduced cardiac reserve and smaller left and right ventricular volumes, as well as decreased vascularity, increased vascular stiffness, and higher pressure of both the pulmonary and systemic vasculature. The purpose of this review is to present major epidemiological evidence linking preterm birth with cardiovascular disease; to discuss findings from clinical studies showing a long-term impact of preterm birth on cardiac remodeling, as well as the systemic and pulmonary vascular systems; to discuss differences across gestational ages; and to consider possible driving mechanisms and therapeutic approaches for reducing cardiovascular burden in individuals born preterm.

Preterm birth (<37 weeks’ gestation) affects over 10% of live births, with rates ranging from 5% to 18% worldwide.^1^ Nearly 85% of these births globally are moderate-to-late preterm (32 to <37 weeks’ gestation), whereas ≈10% are very preterm (28 to <32 weeks’ gestation) and 5% extremely preterm (<28 weeks’ gestation).^1^ Major advances in perinatal care since the 1980s have led to a dramatic increase in survival rates of even the most premature neonates, and now overall survival exceeds 95% in developed countries.^2^ Accordingly, there is a large cohort of preterm-born survivors across gestational age categories now reaching adulthood. In spite of improvements in medical care, preterm-born individuals are at greater risk of all-cause mortality in young adulthood,^3^ as well as cardiometabolic diseases, including type 1 and 2 diabetes mellitus,^4^ hypertension,^5^ pulmonary vascular disease,^6–8^ early heart failure,^9^ and ischemic heart disease.^10^

Preterm birth occurs during a key period of cardiovascular development, leading to an early physiological shift to a relatively hyperoxic environment with increased systemic and decreased pulmonary vascular resistance. As a result, animal and clinical studies across developmental stages have focused on observational and mechanistic insights into cardiovascular changes related to prematurity that may contribute to short- and long-term risk of disease (Figure 1). In this review, we present the latest epidemiological and observational data in humans investigating the cardiovascular impact of being born preterm, broken down into sections covering the cardiac, systemic vascular, and pulmonary vascular systems, highlighting differences in disease risk across gestational ages (Figure 2). We then go on to describe animal and experimental models that have provided mechanistic insights for these unique phenotypic alterations. Given the large proportion of the population born preterm and the strong evidence showing increased long-term cardiovascular risk, the final section of the review provides a framework for considering clinical screening and evaluation.

**Figure 1.**
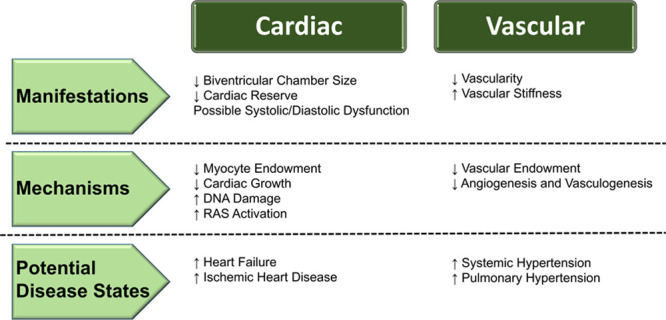
Summary of long-term cardiovascular sequelae of preterm birth. RAS indicates renin-angiotensin system.

**Figure 2.**
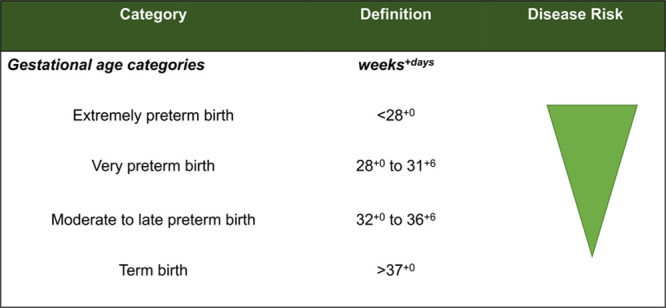
Gestational age categories. Cardiovascular disease risk determined from large population-based studies increases with the degree of prematurity.

## Late Sequelae and Future Cardiovascular Disease Risk

### Early Heart Failure and Young Adult Ischemic Heart Disease Risk: an Independent Effect of Gestational Age?

In 2017, Carr et al^9^ published a register-based cohort study of 2 665 542 individuals born in Sweden from 1987 to 2012 to determine the association between preterm birth and the risk of incident heart failure. Individuals were followed up from 1 year of age until December 31, 2013, with a total follow-up time of 34.8 million person-years and 501 cases of heart failure. After individuals born with potentially confounding malformations were excluded, there were 305 heart failure cases and a follow-up of 34.2 million person-years. Incidence rates of heart failure were inversely related to gestational age at birth, with a 17-fold increased risk in those born extremely preterm compared with those born at term even after adjustment for maternal characteristics, subject sex, birth period, and birth weight for gestational age. While the adjusted risk of incident heart failure was 3.6× higher in subjects born very preterm compared with term, there was no significant increase in the risk of heart failure for subjects born moderate-to-late preterm.

Crump et al^10^ published a similar register-based cohort study in 2019 to investigate the association between prematurity and increased risk of ischemic heart disease in adulthood. A total of 2 141 709 individuals born in Sweden as singleton live births between 1973 and 1994 were included, with follow-up until the end of 2015. The total follow-up time was 30.9 million person-years, with 1921 individuals receiving a diagnosis of ischemic heart disease. Gestational age was inversely associated with ischemic heart disease risk across ages 18 to 43 years, including a strong association among individuals born late preterm. The strength of this relationship was higher in those individuals currently 30 to 43 years of age; interestingly, both preterm birth and early-term birth (37–38 weeks’ gestation) were associated with increased relative risks of ischemic heart disease (53% and 19%, respectively) compared with individuals born full term between 39 to 41 weeks’ gestation.

### Structural and Functional Cardiac Remodeling

The third trimester is a period of rapid cardiac growth, primarily driven by cardiomyocyte hyperplasia. Birth results in a switch in cardiomyocyte growth from a fetal hyperplastic to a neonatal hypertrophic response.^11^ A recent meta-analysis investigating cardiac differences between preterm-born and term-born individuals demonstrated that individuals born preterm have morphological and cardiac functional impairments across all developmental stages including neonatal life, infancy, childhood, and into young adulthood.^12^ These underlying cardiac differences in structure and function that are apparent as early as neonatal life may make the preterm heart more susceptible to heart failure and ischemic heart disease.^13^

The first study to investigate the cardiac impact of being born preterm in young adulthood was done using cardiovascular magnetic resonance (CMR) in 234 individuals, of whom 102 were born preterm with an average gestational age of 30 weeks.^14^ Preterm-born individuals had smaller left ventricular (LV) end-diastolic volumes, smaller internal LV cavity dimensions and lengths, as well as greater LV wall thickness and mass. These cardiac changes were independent of differences in body size and blood pressure between groups. Through the use of a novel computational atlas created from CMR images, it was found that the primary morphological feature contributing to the lower LV end-diastolic volumes was the reduction in LV length. Though the preterm-born young adults had no differences in LV ejection fraction, LV longitudinal systolic strain and strain rate, as well as diastolic strain rate, were reduced. In the same cohort of participants, right ventricular (RV) end-diastolic volumes were also lower and RV mass higher in preterm-born compared with term-born young adults, which could not be accounted for by differences in body size.^15^ Unlike in the LV, RV ejection fraction was lower in the preterm group, with 6% of young adults having values below the clinical normal range (<45%). Preterm-born young adults also had lower RV systolic strain and strain rates, as well as diastolic strain rate.

Similar findings have been reported in an independent cohort of 101 normotensive young adults, of whom 47 were born preterm with an average gestational age of 33 weeks.^16^ Using echocardiography, the study showed that preterm-born young adults had smaller LV end-diastolic volumes and dimensions and increased LV mass indexed to body size, as well as impaired longitudinal systolic strain. In the same cohort, body size indexed RV end-diastolic areas and volumes for echocardiography and CMR were lower in preterm-born individuals,^17^ with RV CMR revealing higher RV mass, lower RV ejection fraction, and a unique 3-dimensional geometry by computational atlas formation in those born preterm. Measurements of RV function by echocardiography, including RV fractional area of change and tricuspid annular plane systolic excursion, were lower in preterm-born compared with term-born young adults, with RV changes remaining significant when adjusting for pulmonary function parameters by spirometry.

**Figure 3.**
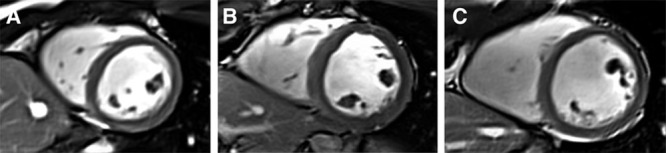
Examples of short-axis end-diastolic cardiac images acquired using cardiovascular magnetic resonance from 3 young adult men born at different gestational ages. A 27-y-old man born at 26^+1^ weeks’ gestation (extremely preterm; **A**); a 27-y-old man born at 32^+5^ weeks’ gestation (moderately preterm; **B**); and a 28-y-old man born at 40^+0^ weeks’ gestation (term; **C**). The extremely preterm–born young adult man (**A**) has the smallest left ventricular (LV) end-diastolic volume index and the lowest LV mass index. Wall thickness is greatest in the moderately preterm–born young adult man (**B**), with visible relative hypertrophy. The young adult man born at term (**C**) has a higher LV end-diastolic volume index than both individuals born preterm, with an LV mass between the two.

Although the findings of reduced LV and RV volumes and dimensions are consistent across studies in adolescence and young adulthood,^14–20^ differences in mass are less consistent. While some studies using both CMR^14,15,17^ and echocardiography^16–18^ have reported higher LV and RV mass in preterm-born young adults compared with term-born controls, others have reported lower mass in those born preterm.^19–21^ A recent study using CMR in a group of 40 normotensive adolescents, of which 20 were born preterm with an average gestational age of 28 weeks, and 70 normotensive young adults, of which 38 were born preterm with an average gestational age of 29 weeks, showed that both LV and RV end-diastolic and stroke volumes were lower in the preterm groups, as was LV mass when indexed to body size.^20^ Interestingly, LV longitudinal systolic strain was higher in the preterm-born young adults in this study, as were RV longitudinal systolic and diastolic strain parameters. Further work is needed to explore these differences, taking into account variability in perinatal characteristics and interventions, as well as lifestyle factors throughout development, such as exercise activity level, socioeconomic status, and diet. How the heart will remodel with the development of other risk factors associated with preterm birth, such as pulmonary and systemic hypertension and obesity,^22^ remains to be determined and may account for some of the variability in results. Part of this variability in hypertrophic response may also be due to changes in perinatal clinical management, which have rapidly evolved in the 1980s and 1990s. Moreover, the most premature individuals, such as those born at <28 weeks’ gestation, may have the lowest cardiac endowment and thus the fewest overall number of cardiomyocytes. As such, cardiac mass may remain lower even if the individual cardiomyocytes are hypertrophied. Individuals born less premature, such as those born around 32 weeks’ gestation, may have the same early hypertrophic stimuli to increase cardiomyocyte size but are also more likely to have a greater total number of cardiomyocytes due to longer in utero development, which may ultimately drive higher mass relative to term-born controls (Figure 3).

The observed underlying changes in cardiac structure and function in preterm-born young adults may reduce their myocardial functional reserve such that they are less able to cope with acute and chronic stressors. Huckstep et al^16^ tested the acute stress response by performing both resting and exercise echocardiography measures with prescribed intensities during exercise based on maximal cardiopulmonary exercise testing. While LV ejection fraction did not differ between the groups at rest, it was significantly lower at 60% intensity and further declined compared with term-born control values at 80% intensity. This impairment in myocardial functional reserve was also a predictor of the widely reported lower peak maximal oxygen consumption and delayed heart rate recovery in preterm-born young adults.^23,24^ Similarly, Goss et al^25^ demonstrated using right heart catheterization that preterm-born young adults had an impaired ability to increase cardiac index and stroke volume from rest to 70% of maximum exercise capacity determined during invasive cardiopulmonary exercise testing. These studies support the idea that preterm-born young adults have impairments in both LV and RV myocardial functional reserve, which is likely to increase susceptibility to heart failure later in life, particularly with additional insults such as hypertension or myocardial infarction.

### Do Systemic Vascular Effects Explain Increased Hypertension Risk in People Born Preterm?

The link between preterm birth and higher blood pressure is now clearly established. Crump et al^5^ showed in a cohort of 636 552 individuals born between 1973 and 1979 followed to age 25 to 37 years that being born preterm results in an increased relative rate of antihypertensive medication prescription, which increased monotonically with the degree of prematurity and was independent of fetal growth restriction. Recent meta-analyses showed that preterm-born adolescents and young adults had a systolic blood pressure of +3.4 to 4.2 mm Hg and diastolic blood pressure of +2.1 to 2.3 mm Hg compared with term-born peers.^26,27^ Interestingly, women seem more affected than men.^27^ A population-based study further showed that women born preterm, particularly if they were born at <32 weeks’ gestation, have an increased risk of pregnancy-induced hypertension.^28^

Preterm birth may disrupt or even prematurely arrest development of the vasculature, impacting both vessel structure and organogenesis.^29^ Abnormal placentation and altered in utero vascular remodeling and angiogenesis are also commonly associated with preterm delivery^30^ and may lead to abnormal vascular development in the offspring. Furthermore, interrupted vascular tree development is a key component of several major prematurity-related complications, including bronchopulmonary dysplasia and retinopathy of prematurity.^31^ Growth of the aorta is reduced in preterm infants compared with intrauterine development.^29^ Overall, it can be postulated that preterm birth results in a restricted vascular bed, narrowed and stiffer arteries, all predisposing to endothelial dysfunction and arterial hypertension.

**Figure 4.**
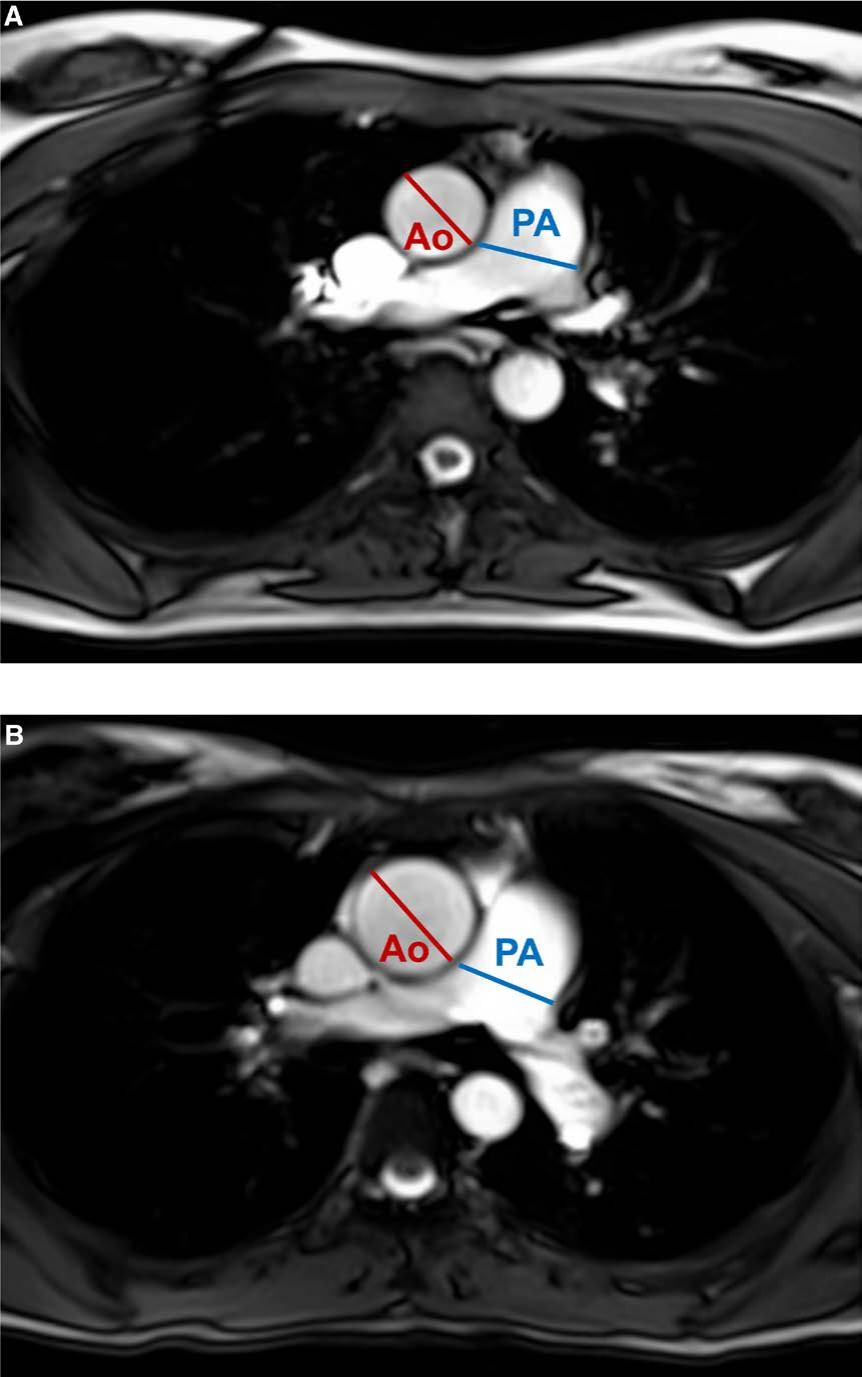
Examples of axial cardiovascular magnetic resonance images at the level of the pulmonary artery (PA) bifurcation from 2 young adult women born at different gestational ages demonstrating increased PA to aorta (Ao) after preterm birth. A 28-y-old woman born at 27^+6^ weeks’ gestation (extremely preterm; PA:Ao is 1.03; **A**) and a 28-y-old woman born at 40^+2^ weeks’ gestation (term; PA:Ao is 0.79; **B**).

In several studies in young adults born extremely preterm, the lumen of the ascending, thoracic, and abdominal aorta was shown to be smaller (Figure 4).^19,32,33^ For the thoracic and abdominal aorta, these differences appear to remain even after taking into account body size.^34^ Data on other major arteries, such as the carotid and brachial arteries, are less consistent, with studies showing no differences or smaller internal diameters.^35,36^ Interestingly, adjustment for body size has shown to diminish or remove group differences in the size of conduit arteries,^37^ with even slightly larger brachial cross-sectional areas observed after adjustment in young adults born preterm.^34^

Arterial stiffness has been investigated in preterm-born individuals using surrogate indices such as pulse wave velocity (PWV; reflective of conduit arterial stiffness), augmentation index (the reflection pressure wave modulated by vessel stiffness and blood pressure), and other measures of vascular elasticity. Arterial distensibility and elasticity depend largely on the ratio of elastin and the more rigid collagen in arterial walls. In humans, elastin synthesis peaks toward the end of gestation and continues into early childhood, declining rapidly thereafter, with minimal synthesis by adulthood.^38^ Disruption of elastin synthesis at the end of gestation or with preterm birth may increase arterial stiffness as supported by experimental models.^39,40^ Desmosine levels—an elastin breakdown product—were also elevated in a cohort of young adults born extremely preterm, though they did not correlate with PWV or augmentation index.^41^ Boardman et al^34^ performed a detailed study of major systemic arteries in 102 young adults born very preterm at an average of 30 weeks’ gestation compared with 102 term-born age-matched controls. Preterm-born young adults had increased carotid-femoral PWV, evaluated using 3 different methods, as well as increased augmentation index that was inversely correlated with the smaller aortic luminal area in the preterm group. However, after taking into account blood pressure in the analysis, the differences were markedly reduced. It is also possible that body mass index, which was higher in the preterm-born compared with term-born subjects, could explain a proportion of the differences between groups as preterm-born individuals are more likely to be obese^22^ and obesity associates with increased arterial stiffness from adolescence to adulthood.^42^ In adolescents and adults born late preterm, carotid-femoral PWV was similar to term-born controls, whereas carotid-radial PWV was marginally but significantly increased, despite higher systolic blood pressure levels.^43,44^ In a group born more prematurely (average 26 gestational weeks), Kowalski et al^19^ reported similar carotid-radial PWV and augmentation index compared with term-born controls, despite smaller aortic diameters, higher blood pressures, and no difference in body mass index between groups. However, in a recent study by Barnard et al,^45^ aortic PWV was increased in 10 very preterm-born young adults compared with 12 term-born blood pressure and body mass index–matched controls.

Although a single study suggests diminished endothelial function in 32 young adults born extremely preterm,^46^ the majority of studies performed in adolescents and young adults born very or extremely preterm have not shown any alteration in endothelial function associated with preterm birth evaluated using brachial artery flow–mediated dilation or reactive hyperemia index.^19,35,36^ Studies reporting carotid intima-media thickness—an early marker of atherosclerosis and nonatherosclerotic remodeling—in preterm-born adolescents and adults have consistently shown no differences compared with term-born controls.^19,36^ In line with this, Hovi et al^36^ found that carotid intima-media thickness did not differ between very preterm and term-born adults, but because lumen diameter was much smaller in the preterm group, carotid intima-media thickness was increased relative to lumen diameter.

At the microvascular level, Kistner et al^47^ reported that, independent of retinopathy of prematurity, retinal vascular caliber and density were decreased in children and young adults born very preterm on average at 30 weeks. Furthermore, skin microvascular perfusion and density have been shown to be reduced and to correlate with higher blood pressure and circulating antiangiogenic factors.^32,48^ Reduced systemic angiogenic capacity could underlie impaired vascular growth. Neonatal exposure to high oxygen, which mimics preterm birth–related physiological conditions, reduced in vitro angiogenic capacity and systemic microvascular density in adult rats.^49,50^ Supporting this possibility, endothelial colony–forming cells collected from venous blood had altered in vitro proliferation and angiogenic capacity in young adults born extremely preterm, which correlated with increased blood pressure.^51^ Further work is needed to clarify the role of defective vascular tree development or repair and related mechanisms, such as epigenetic changes and accelerated senescence of progenitor cells,^52^ contributing to the development of cardiovascular diseases in individuals born preterm.

Beyond altered vascular structure and growth, other elements may contribute to the etiology of hypertension in the preterm-born population. Studies of children and adults born very or extremely preterm have shown that preterm birth is associated with altered nephrogenesis and smaller kidneys, with the reduction in kidney size correlating with higher blood pressure in adults.^53^ Studies have also shown higher hemoglobin,^54^ evidence of sympathetic nervous autonomic system activation,^55^ and hypothalamus-pituitary-adrenal dysregulation^56^ in preterm-born adults, which all potentially contribute to higher blood pressure. A recent study of 101 young adults born on average at 27 weeks’ gestation compared with 105 young adults born at term found no association between preterm birth and markers of inflammation and oxidative stress, as well as no association between high blood pressure and other cardiovascular risk factors (such as glucose intolerance, which is also more prevalent in preterm-born individuals), suggesting a unique mechanism to hypertension and disease development.^57^ Perinatal interventions and complications that are common in preterm infants, including perinatal infection, accelerated postnatal weight gain, parenteral nutrition, and antenatal and postnatal steroids, have been shown to relate to increased vascular stiffness and may contribute to long-term cardiovascular remodeling.^58^ Further work is needed to look at the long-term effects of these early life interventions. In addition, the hereditary component of hypertension among individuals born preterm has not yet been fully investigated. Indeed, women who had preeclamptic pregnancies (an antecedent to 20%–30% of preterm births) or who had spontaneous preterm deliveries were recently shown to be at increased risk of future cardiovascular diseases.^59^ Whether genetically inherited factors add to the risk of systemic hypertension related to altered organ and vascular development and function as a result of being born preterm is plausible but remains to be addressed.

### Risk of Pulmonary Vascular Disease

In addition to the direct cardiac and systemic vascular effects of premature birth, individuals born preterm are at increased risk for pulmonary vascular disease as evidenced by both epidemiological and clinical data. The initial epidemiological data supporting prematurity as a risk factor for adult pulmonary hypertension come from a case-control study of adults in the Swedish pulmonary arterial hypertension registry. When compared with controls from the birth register, matched by birth year and hospital, premature birth was associated with an odds ratio of 3.08 for adult pulmonary arterial hypertension.^6^ This relationship persisted after adjusting for known risk factors such as congenital heart disease and bronchopulmonary dysplasia. Further, the association was even stronger in children and adolescents (odds ratio, 8.46), likely reflecting improved neonatal survival of the most at-risk individuals.^7,8^

Recent clinical studies demonstrate the presence of mild pulmonary hypertension in a significant percentage of adolescents and young adults born moderately to extremely premature. Among 193 adolescents with an average gestational age of 27 weeks, nearly half had a mean pulmonary artery pressure by echocardiogram >20 mm Hg.^60^ Additional echocardiogram-based studies in adolescents and adults born preterm have reported an elevated tricuspid regurgitation velocity and lower pulmonary artery acceleration times in those born preterm, indicating increased pulmonary vascular resistance relative to term-born age-matched controls.^16,19,61^ The only right heart catheterization study conducted to date enrolled 11 young adults born preterm with an average gestational age of 28 weeks, of whom 5 of 11 satisfied criteria for pulmonary hypertension as defined by a mean pulmonary artery pressure >20 mm Hg. Furthermore, pulmonary vascular resistance and arterial elastance were significantly elevated among preterm adults, suggesting a stiffer pulmonary vascular bed overall.^25^ Among these subjects, RV-pulmonary vascular coupling was impaired in preterm-born relative to term-born subjects.^62^ In contrast, an echocardiography-based study of ventriculo-vascular coupling in young adults born preterm with an average gestational age of 33 weeks demonstrated preserved RV coupling relative to term-born adults.^16^ The lower gestational age in the catheter-based study further suggests that early RV-pulmonary artery uncoupling may be gestational age dependent. Further, the degree of uncoupling was unexpected for the mild elevation in afterload and supports the concept of prematurity serving as a direct insult to the RV as well. In summary, changes in RV function and pulmonary vascular resistance are present across gestational ages, and it is possible that RV-pulmonary vascular uncoupling will occur sooner even in those born moderate-to-late preterm, warranting further follow-up into mid and late adulthood.

Several studies have assessed the pulmonary vascular response to exercise or hypoxia. In an echocardiography-based exercise study, adults born premature demonstrated an exaggerated increase in estimated pulmonary pressure.^63^ These findings, however, were not confirmed in the aforementioned right heart catheterization–based study, possibly due to smaller sample size.^25^ Among the studies that have assessed the response to hypoxia, the hypoxic vasoconstrictor response appears intact and similar between term- and preterm-born subjects.^25,61,63^ Some of these studies have identified bronchopulmonary dysplasia as one of the possible key drivers of overt late pulmonary vascular disease, though it is unlikely to be the sole contributor.^25,60^

Finally, although the overall severity of pulmonary vascular disease appears mild in childhood and adult studies, a subset of premature infants develop more severe pulmonary vascular disease. Additional studies are needed to understand the natural history of pulmonary vascular-RV development in these high-risk individuals. In the meantime, even small elevations in mean pulmonary artery pressure are associated with a significant increase in all-cause mortality in adults.^64,65^ Therefore, the lifetime impact may be significant even in relatively healthy individuals with a history of preterm birth.

## Possible Mechanisms and Therapeutic Approaches

Mechanistic studies of the cardiac and vascular sequelae of premature birth are somewhat constrained due to limitations of available animal models. Whereas both small and large animal models can be delivered prematurely, there is limited-to-no ability to support the degree of prematurity that is encountered and survivable in humans. However, both may be informative regarding specific aspects of cardiovascular development and will be discussed here.

Rodent models have been used for decades to study the sequelae of premature birth, specifically as related to the development of bronchopulmonary dysplasia. Although models differ in the duration and severity, most utilize postnatal hyperoxia exposure to recapitulate the alveolar and vascular oversimplification characteristic of bronchopulmonary dysplasia.^66^ The application of oxygen exposure in this model aims to mimic the relative hyperoxic environment common to premature birth. Birth results in an immediate transition to normoxia, which is relatively hyperoxic compared with normal fetal life. Although hyperoxic animal models do not recapitulate prematurity per se, they do mimic the developmentally inappropriate relative hyperoxia that a preterm infant experiences and is highly relevant to the developing heart. Specifically, at birth, the rodent heart is predominantly mononucleated, with terminal cell cycle arrest and transition from hyperplasia to hypertrophy for cellular growth occurring during the first week postnatally.^67^ When hyperoxia is applied, cell cycle arrest is accelerated through activation of DNA damage responses.^68^

Postnatal hyperoxia exposure in rodents has been utilized to recapitulate both the late RV-pulmonary vascular and LV-systemic vascular complications of premature birth. Animals develop moderate-to-severe pulmonary hypertension in the neonatal period, with some ability to recover in adolescence and adulthood.^69^ There appears to be some degree of pulmonary revascularization, only to have accelerated vascular pruning with aging.^70^ Pulmonary vascular disease is associated with bimodal RV dysfunction, with early uncoupling and RV dysfunction due to the severity of the vascular disease. There is a period of cardiac recovery, as the pulmonary hypertension improves, followed by late RV failure related to emergence of mitochondrial dysfunction and accumulation of mitochondrial DNA damage.^69,71^ Mitochondrial oxidation also drives cardiomyocyte loss beginning in the neonatal time period and may be rescued with administration of antioxidants such as mitoTEMP.^68,72^ Therefore, a diminished cardiomyocyte endowment may further contribute to cardiac dysfunction. In the LV, postnatal hyperoxia results in remodeling and impaired systolic function even before systemic blood pressure elevation, including an increase in LV cardiomyocyte hypertrophy, fibrosis, and in markers of premature senescence.^73^ Moreover, these early cardiac alterations increased the susceptibility of rodents to develop heart failure following pressure overload induced by angiotensin II infusion. Development of cardiac alterations in adulthood, including fibrosis and hypertrophy, was linked to upregulation of angiotension II receptors type 1 and 2 in the LV, signifying increased renin-angiotensin system activation, which was prevented with early treatment with the AT1 (angiotensin II type 1) receptor antagonist losartan and with an antagonist of the Toll-like receptor-4 proinflammatory pathway.^74,75^ This indicates that a number of deleterious mechanisms for myocardial development are triggered by preterm birth–related conditions along with neonatal oxidative injury.

In an ovine model of preterm birth (14 days premature), LV and RV cardiomyocytes were hypertrophied in the preterm-born lambs compared with controls at 9 weeks postnatal age.^76^ It is noteworthy that preterm-born sheep also had an ≈7-fold increase in interstitial collagen deposition, even without the normal inflammatory and stress-related conditions that are commonly associated with preterm birth in humans.^77^ Nucleation and ploidy of the cardiomyocytes were also shown to be altered, supporting the argument that there are irreversible myocardial stress–related changes in DNA in the preterm-born lambs. At 14.5 months postnatal age follow-up, preterm-born sheep had thinner RV walls, a higher reserve of immature undifferentiated cardiomyocytes, lower cardiomyocyte numbers, and smaller cardiomyocyte areas with reductions in RV functional and adaptive capacity.

Currently, there are limited data to suggest specific therapeutic strategies in adults born preterm to treat hypertension or modify heart failure risk. Elevated plasma levels of angiotensin I and the presence of autonomic dysfunction and impaired heart rate recovery may suggest a potential role for angiotensin or adrenergic blockade, but these have not been specifically evaluated in preterm subjects.^23,24,78^ There is evidence that neonatal risk factors do appear to be modifiable. For example, exclusive human milk feeding during infancy, as compared with formula, was associated with larger LV and RV end-diastolic and stroke volumes and smaller pulmonary artery to aortic diameter ratios in adulthood.^79^ Ultimately, further study is needed to understand the role of intervention strategies throughout the life span, including lifestyle modifications such as exercise and pharmacological interventions, on improving cardiac remodeling and decreasing long-term cardiovascular risk.

## Conclusions: Call to Arms Regarding Awareness and Screening

**Figure 5.**
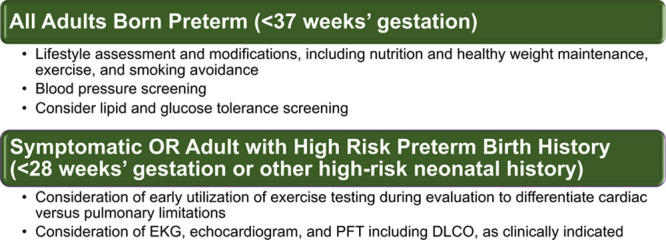
Screening considerations for adults born preterm. DLCO indicates diffusing capacity of the lungs for carbon monoxide; and PFT, pulmonary function test.

It is now clear that preterm birth is associated with increased risk of cardiovascular diseases including ischemic heart disease and hypertension, as well as increased risk of heart failure. The mechanisms are not completely understood, and current evidence indicates that the disruption of heart, vessels, and overall organ development associated with preterm birth contributes to disease risk, most likely in addition to hereditary components. While much remains to be unraveled regarding mechanisms, attention should now also be directed on how to effectively prevent hypertension and disease occurrence, from the neonatal period through adult life. Subclinical diseases, including high blood pressure and mild cardiac dysfunction, are detectable in young adulthood and might not be accompanied by classically associated risk factors such as obesity. Thus, recognition of preterm birth as a significant risk factor for cardiovascular disease becomes imperative for clinicians, parents, and the preterm-born adults. Birth history should be included in best practice guidelines from childhood to adulthood to permit healthy lifestyle counseling and appropriate screening. Among those symptomatic or at the highest risk, clinicians should consider that symptoms may be multifactorial, including a compilation of cardiac, pulmonary, and vascular sequelae of prematurity, and may require more nuanced testing and clinical evaluation, such as early utilization of exercise testing (Figure 5). Modifying the lifetime cardiovascular risk for this population will require multidisciplinary collaboration across ages and organ systems, from neonatologists to internists including cardiologists, pulmonologists, as well as our preterm-born patients themselves.

## Sources of Funding

A.J. Lewandowski is funded by a British Heart Foundation Intermediate Basic Science Research Fellowship (BHF FS/18/3/33292). A.M. Nuyt is funded by the Canadian Institutes of Health Research (CIHR 399001 and 313082), the Heart and Stroke Foundation of Canada, the Canadian Foundation for Innovation, and the Cercle Sainte-Justine Developmental Origins of Health and Diseases Research Chair. K.N. Goss is funded by a Parker B. Francis Fellowship Award and American Heart Association Career Development Award (K.N. Goss, No. 18CDA34110440).

## Disclosures

None.
